# D-cysteine ethyl ester and D-cystine dimethyl ester reverse the deleterious effects of morphine on arterial blood-gas chemistry and Alveolar-arterial gradient in anesthetized rats

**DOI:** 10.1016/j.resp.2022.103912

**Published:** 2022-04-18

**Authors:** Paulina M. Getsy, Alex P. Young, Alan Grossfield, James M. Seckler, Christopher G. Wilson, Benjamin Gaston, James N. Bates, Stephen J. Lewis

**Affiliations:** a Department of Pediatrics, Case Western Reserve University, Cleveland, OH 44106, USA; b Pediatric Respiratory Medicine, University of Virginia School of Medicine, Charlottesville, VA 22908, USA; c Department of Biochemistry and Biophysics, University of Rochester Medical Center, Rochester, NY 14642, USA; d Department of Biomedical Engineering, Case Western Reserve University, Cleveland, OH 44106, USA; e Basic Sciences, Division of Physiology, School of Medicine, Loma Linda University, Loma Linda, CA 92350, USA; f Herman B Wells Center for Pediatric Research, Indiana University, School of Medicine Indianapolis, IN 46202, USA; g Department of Anesthesiology, University of Iowa Hospitals and Clinics, Iowa City, IA 52242, USA; h Department of Pharmacology, Case Western Reserve University, Cleveland, OH 44106, USA; i Functinal Electrical Stimulation Center, Case Western Reserve University, Cleveland, OH 44106, USA

**Keywords:** Morphine, D-cysteine ethyl ester, D-cystine dimethyl ester, Arterial blood gases, Anesthetized rats

## Abstract

We determined whether intravenous injections of the membrane-permeable ventilatory stimulants, D-cysteine ethyl ester (ethyl (2 S)– 2-amino-3-sulfanylpropanoate) (D-CYSee) and D-cystine dimethyl ester (methyl (2 S)– 2-amino-3-[[(2 S)– 2-amino-3-methoxy-3-oxopropyl]disulfanyl] propanoate) (D-CYSdime), could overcome the deleterious actions of intravenous morphine on arterial blood pH, pCO_2_, pO_2_ and sO_2_, and Alveolar-arterial (A-a) gradient (i.e., the measure of exchange of gases in the lungs) in Sprague Dawley rats anesthetized with isoflurane. Injection of morphine (2 mg/kg, IV) caused pronounced reductions in pH, pO_2_ and sO_2_ accompanied by elevations in pCO_2_, all which are suggestive of diminished ventilation, and elevations in A-a gradient, which suggests a mismatch of ventilation-perfusion. Subsequent boluses of D-cysteine ethyl ester (2 × 100 μmol/kg, IV) or D-cystine dimethyl ester (2 ×50 μmol/kg, IV) rapidly reversed of the negative actions of morphine on pH, pCO_2_, pO_2_ and sO_2_, and A-a gradient. Similar injections of D-cysteine (2 × 100 μmol/kg, IV) were without effect, whereas injections of D-cystine (2 × 50 μmol/kg, IV) produced a modest reversal. Our data show that D-cysteine ethyl ester and D-cystine dimethyl ester readily overcome the deleterious effects of morphine on arterial blood gas (ABG) chemistry and A-a gradient by mechanisms that may depend upon their ability to rapidly enter cells. As a result of their known ability to enter the brain, lungs, muscles of the chest wall, and most likely the major peripheral chemoreceptors (i.e., carotid bodies), the effects of the thiolesters on changes in ABG chemistry and A-a gradient elicited by morphine likely involve central and peripheral mechanisms. We are employing target prediction methods to identify an array of in vitro and in vivo methods to test potential functional proteins by which D-CYSee and D-CYSdime modulate the effects of morphine on breathing.

## 1. Introduction

Administration of opioids to humans exerts deleterious effects on the status of arterial blood gas chemistry by suppressing breathing and impairing gas-exchange in alveoli (Rybro et al., 1982; Cepeda et al., 2003; Chow et al., 2003; Cashman and Dolin, 2004; Taylor et al., 2005; Wang et al., 2005; Lötsch et al., 2006; Dahan et al., 2010). Opioids elicit effects in animals that impair arterial blood gas (ABG) chemistry via cardiopulmonary afferent- and centrally-induced decreases in breathing, enhanced rigidity of skeletal muscle in the chest-wall/abdomen, and increased resistance of the upper airway via collapse/closure of the larynx and vocal folds (Willette et al., 1982, 1983, 1987; Bennett et al., 1997; Niedhart et al., 1989; Campbell et al., 1995; Bowdle, 1998; Kaczyńska and Szereda-Przestaszewska, 2005; Dahan et al., 2010). Additionally, opioids have deleterious actions on ventilation-perfusion in the lungs (Ling et al., 1985; Szikszay et al., 1986; Copland et al., 1987; Hannon and Bossone, 1991; Shafford and Schadt, 2008; Henderson et al., 2014; Jenkins et al., 2021; Gaston et al., 2021), and depress activity of carotid body chemoreceptor afferents and their ability to (1) respond to environmental hypoxia and/or hypercapnia (McQueen and Ribeiro, 1980, 1981; Zimpfer et al., 1983; Kirby and McQueen, 1986; Mayer et al., 1989), and (2) diminish changes in ventilation during hypoxic and/or hypercapnic gas challenges (Zhang et al., 2009; May et al., 2013a; b).

Because of evidence that morphine reduces intracellular levels of reducing agents and shifts the redox state to a higher oxidative status (Macchia et al., 1999; Polanco et al., 2009), we reasoned that rapidly elevating intracellular levels of the endogenous reducing agent, L-cysteine, may modulate the morphine-induced depression of breathing (Mendoza et al., 2013). We used the highly cell-penetrant thiolester, L-cysteine ethyl ester (L-CYSee) (Fukui et al., 1994; Clancy et al., 2001), which rapidly penetrates into peripheral and central structures (Servin et al., 1988) thereby increasing intracellular concentrations of cysteine (Hobbs et al., 1993; Deneke, 2000) via the actions of a carboxyl-esterase enzyme (Butterworth et al., 1993). Mendoza et al. (2013), and determined that intravenous injection of L-CYSee elicited a rapid and sustained reversal of the deleterious actions of morphine on ABG chemistry and Alveolar-arterial (A-a) gradient (i.e., L-CYSee reversed the detrimental effects on the state of ventilation-perfusion in the lungs) in tracheotomized isoflurane-anesthetized rats, but not in those without a tracheotomy. As such, L-CYSee overcame the deleterious effects of morphine on ventilation, but its ability to raise resistance in the upper airway impairs the processes in the lungs that regulate gas-exchange.

We now report that D-cysteine ethyl ester (D-CYSee) or D-cystine dimethyl ester (D-CYSdime) elicit immediate and enduring reversal of the deleterious actions of morphine on ABG chemistry and A-a gradient in rats under isoflurane anesthesia without the need for tracheotomy. As such, the processes by which L- and D-CYSee overcome opioid-induced respiratory depression are independent of stereospecific processes, whereas the ability of L-CYSee to induce obstruction/collapse of upper airway structures involves stereospecific mechanisms not activated by D-CYSee or by D-CYSdime.

## 2. Materials and Methods

### 2.1. Rats and surgeries

All studies were carried out in accordance with the National Institutes of Health Guide for the Care and Use of Laboratory Animals (NIH Publication No. 80–23) revised in 1996. The protocols were approved by the Animal Care and Use Committee of the University of Virginia. Adult male Sprague Dawley rats were purchased from Harlan (Madison, WI). The numbers of rats in each morphine study group and their body weights are shown in Table 1. Each group consisted of 5 rats and there were no differences in body weights between the groups in each of the three studies (P > 0.05, for all between-group comparisons). The rats were subjected to 2% isoflurane anesthesia delivered in room air (21% O_2_) and upon transferring to a surgical table, anesthesia was maintained via a face mask, which delivered isoflurane (2%) in room-air. The body temperatures of the rats were kept at 37.2 ± 0.2°C via a thermometer placed 4–5 cm into the rectum that was connected to a heating pad controlled by input from the thermometer. Fluid-filled catheters were then placed into a femoral vein and femoral artery (Mendoza et al., 2013, 2014). The wounds were then sutured closed and anesthesia was maintained at 1.5% isoflurane in room-air. The anesthetic and surgical procedures were all completed in less than 30 min in each rat.

### 2.2. Blood gas measurements and determination of Alveolar-arterial gradient

Samples (approximately 120 μL) of arterial blood were removed from the rats at pre-determined time points during the experiments (Table 2) as detailed in previous reports (Mendoza et al., 2013; Henderson et al., 2014; Gaston et al., 2021; Jenkins et al., 2021). Arterial blood pH, pCO_2_, pO_2_and sO_2_ values were determined via an ABL800 FLEX blood-gas analyzer (Radiometer, Denmark). The A-a gradient is a calculated measure of O_2_ partial pressure differences between those in the alveoli and those in arterial blood (Torda, 1981; Stein et al., 1995; Story, 1996; Mendoza et al., 2013). More specifically, A-a gradient is equal to PAO_2_ - PaO_2_, in which PAO_2_ is alveolar O_2_ partial pressure, and PaO_2_ is the measured partial pressure of O_2_ in arterial blood. PAO_2_ is calculated by the formula, [(FiO_2_ x (P_atm_-P_H2O_) - (PaCO_2_/RE)], in which FiO_2_ is the relative fraction of O_2_ within the inspired air; P_atm_ is pressure in the atmosphere; P_H2O_ is the partial pressure of H_2_O within the inspired air; PaCO_2_ is the partial pressure of arterial blood CO_2_ and RE (respiratory gas exchange ratio) is taken as the ratio of CO_2_ eliminated divided by the amount of O_2_ consumed. We employed the value of room-air FiO_2_ to be 21% = 0.21, the P_atm_ value to be 760 mmHg, and the P_H2O_ value to be 47 mmHg (Henderson et al., 2014). RE values were not calculated directly, but we used a resting RE value of 0.9 in our adult male rats on the basis of reports by other groups (Stengel et al., 2010; Chapman et al., 2012; Gaston et al., 2021). In accordance with studies detailed by Mendoza et al. (2013) we employed a RE value of again 0.9 to calculate A-a gradients following the injections of morphine and thiolesters, since morphine (Hauner et al., 1988; Lelevich, 2011) and we assume thiolesters (Mendoza et al., 2013) do not directly affect RE. In the present study, it is most likely that we had a mismatch of ventilation-perfusion as well as alveolar hypoventilation. Usually, when these two events occur together and can be overcome by drug therapy, for example, the cause is decreased minute ventilation that causes atelectasis (Mendoza et al., 2013; Gaston et al., 2021). A decrease in PaO_2_, without change in A-a gradient is usually associated by an increase in PaCO_2_ if caused by diminished ventilation. An elevated A-a gradient is most often due to limitation of O_2_ diffusion that is often not able to be reversed, or a mismatch of ventilation-perfusion processes (Gaston et al., 2021).

### 2.3. Protocols

The protocols for the morphine studies are described in Table 2. The 2 mg/kg dose of morphine was chosen because it elicits robust changes in ABG chemistry in isoflurane-anesthetized rats while not causing lethal respiratory depression (Mendoza et al., 2013). A blood sample was taken from all rats 15 min before any injections were given. All of the following rats were injected with morphine (2 mg/kg, IV) and arterial blood samples were taken after 15 min to evaluate changes in ABG chemistry and A-a gradient. Within seconds of blood withdrawal, the rats received injection 1 of the test agent; **Study A:** vehicle (saline, 100 μL/100 g body weight) or D-CYSee (100 μmol/kg); **Study B:** vehicle (saline, 100 μL/100 g) or D-CYSdime (50 μmol/kg; n = 5 rats, 323 ± 2 g), and **Study C:** vehicle (saline, 100 μL/100 g body weight), or D-cysteine (100 μmol/kg) or D-cystine (50 μmol/kg). A blood-gas sample was taken 15 min afterwards (i.e., 30 min post-injection of morphine). After 15 min (i.e., 45 min post-injection of morphine), the rats received injection 2 of vehicle, D-CYSee (100 μmol/kg), D-CYSdime (50 μmol/kg), D-cysteine (100 μmol/kg) or D-cystine (50 μmol/kg). Another arterial blood sample was taken 15 min after the second injections (60 min post-morphine). To obtain time-matched control data, arterial blood samples were taken at the above times in another group of rats (n = 5 rats, 321 ± 3 g) that just received an injection of vehicle (saline, 100 μL/100 g body weight) at injection times described above.

### 2.4. Drugs

A liquid injectable form of (+)-morphine sulfate (10 mg/ml) was obtained from Baxter Healthcare Corporation (Deerfield, IL, USA). D-cysteine hydrochloride monohydrate and D-cystine dihydrochloride dihydrate were obtained from Sigma-Aldrich (St. Louis, MO, USA). D-cysteine ethyl ester was from ChemImpex (Wood Dale, IL, USA). D-cystine dimethyl ester dihydrochloride was obtained from Chemcruz (Dallas, TX, USA). Isoflurane was from Piramal Critical Care Inc. (Bethlehem, PA, USA).

### 2.5. Statistics

All data points are shown as mean ± SEM. The data were subjected to one-way or two-way ANOVA and the Student’s modified *t* test with Bonferroni corrections for multiple comparisons between means utilizing the error mean square terms generated by the ANOVA analyses (Wallenstein et al., 1980) as described in full by Gaston et al. (2021). A value of *P* < 0.05 was taken to denote statistical significance.

## 3. Results

### 3.1. Changes in ABG chemistry and A-a gradient elicited by morphine

**Fig. 1.**
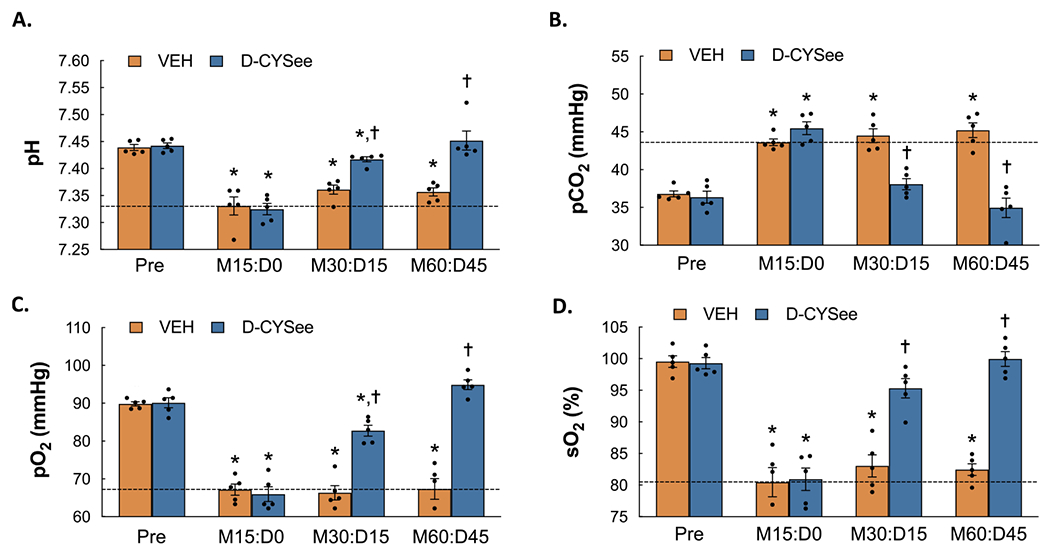
Arterial blood-gas chemistry (pH, pCO_2_, pO_2_, SO_2_) values prior to administration of morphine (Pre), 15 min after injection of morphine sulfate (2 mg/kg, i.v.) (M15:D0) and subsequently 15 min after each of two injections of vehicle or D-cysteine ethyl ester (D-CYSee, 100 μmol/kg, iv) (M30:D15 and M60:D45, respectively) in isoflurane-anesthetized rats. **Panel A:** pH. **Panel B:** pCO_2_. **Panel C:** pO_2_. **Panel D:** sO_2_. The data are presented as mean ± SEM. There were 5 rats in each group. * *P* < significant difference from Pre values. ^†^*P* < 0.05, D-CYSee versus vehicle.

**Fig. 2.**
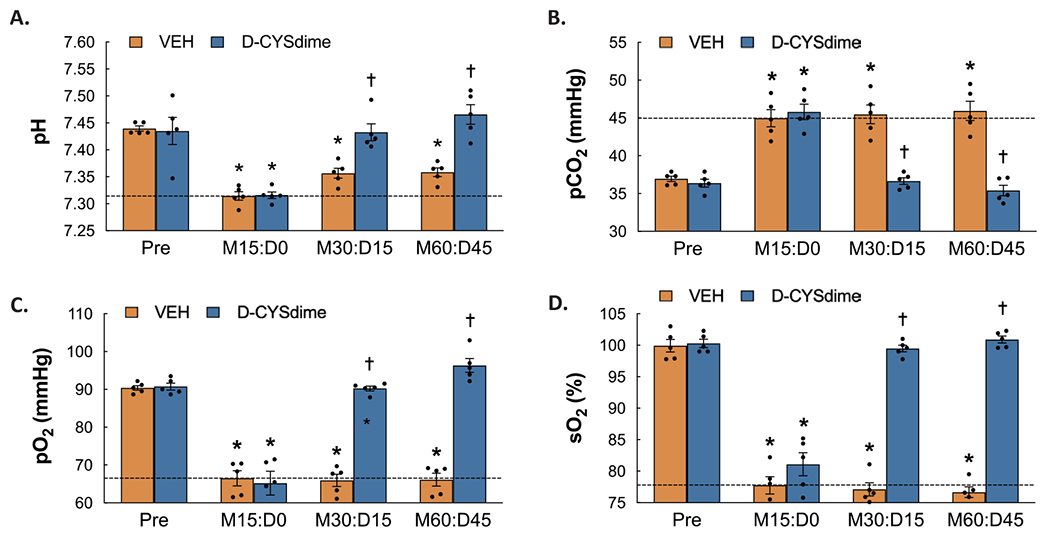
Arterial blood-gas chemistry values prior to administration of morphine (Pre), 15 min after injection of morphine sulfate (2 mg/kg, i.v.) (M15:D0) and subsequently 15 min after each of two injections of vehicle or D-cystine dimethyl ester (D-CYSdime, 100 μmol/kg, iv) (M30:D15 and M60:D45, respectively) in isoflurane-anesthetized rats. **Panel A:** pH. **Panel B:** pCO_2_. **Panel C:** pO_2_. **Panel D:** sO_2_. The data are presented as mean ± SEM. There were 5 rats in each group. * *P* < significant difference from Pre values. ^†^*P* < 0.05, D-CYSdime versus vehicle.

**Fig. 3.**
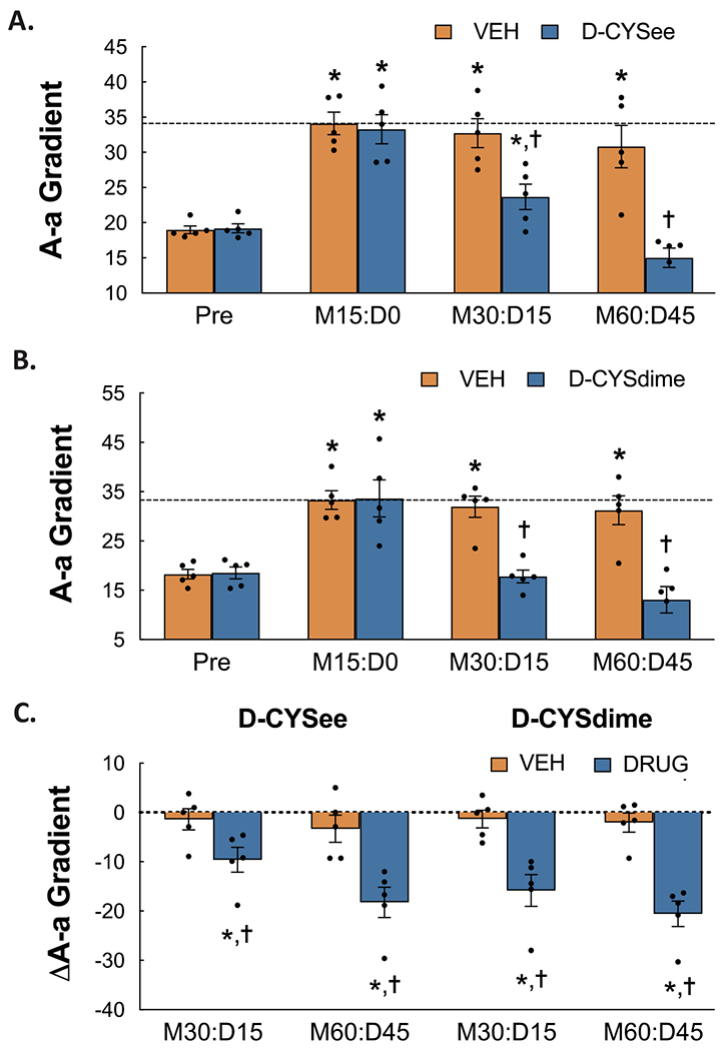
Alveolar-arterial (A-a) gradient values prior to administration of morphine (Pre), 15 min after injection of morphine sulfate (2 mg/kg, i.v.) (M15:D0) and subsequently 15 min after each of two injections of vehicle or D-cysteine ethyl ester (D-CYSee, 100 μmol/kg, iv) (**Panel A**) or vehicle or D-cystine dimethyl ester (D-CYSdime, 100 μmol/kg, iv) (**Panel B**) (M30:D15 and M60:D45, respectively) in isoflurane-anesthetized rats. **Panel C** shows the arithmetic changes in A-a gradient from the morphine values elicited by injections of vehicle, D-CYSee, or D-CYSdime. The data are presented as mean ± SEM. There were 5 rats in each group. * *P* < significant difference from Pre values. ^†^*P* < 0.05, D-CYSdime versus vehicle.

Resting values before injection of morphine for pH, PCO_2_, PO_2_, SO_2_ and A-a gradient were similar to one another in all groups of rats (Figs. 1–3, Table 4; *P* > 0.05, for all between group comparisons). Recorded values for pH, pCO_2_, pO_2_, SO_2_ and A-a gradient remained constant throughout a control study in which rats received injections of vehicle only (Table 3). Injection of morphine elicited sustained changes in ABG chemistry and A-a gradient. These changes included falls in pH, pO_2_ and sO_2_ values that were associated with elevated levels of pCO_2_ and A-a-gradient (Figs. 1–3, Table 4). The morphine-induced changes in ABG chemistry and A-a gradient were equivalent in all groups (*P* > 0.05, for all between-group comparisons).

### 3.2. Reversal of the morphine-induced changes in ABG chemistry by D-CYSee

ABG chemistry values before and after injection of morphine (2 mg/kg/kg, IV) and after 2 subsequent injections of D-CYSee (100 μmol/kg, IV) are shown in Fig. 1. As seen in **Panel A**, the injection of morphine induced sustained reductions in pH from Pre values in rats that were injected with vehicle 30 min after administration of morphine (values are those 15 min after injection of vehicle, **M30:D15**) and again 60 min after administration of morphine (values are those 15 min after the second injection of vehicle, which occurred at time point 30 min, **M60: D45**). As also seen in **Panel A**, the first and second injections of D-CYSee caused a pronounced recovery of pH. As seen in **Panel B**, the sustained increases in CO_2_ produced by morphine in vehicle-injected rats were markedly diminished in rats that received injections of D-CYSee. As seen in **Panels C** and **D**, the sustained decreases in pO_2_ and SO_2_ elicited by morphine in vehicle-injected rats were markedly diminished in rats that received injections of D-CYSee.

### 3.3. Reversal of the morphine-induced changes in ABG chemistry by D-CYSdime

ABG chemistry values recorded before and after injection of morphine (2 mg/kg/kg, IV) and following two injections of D-CYSdime (50 μmol/kg, IV) are summarized in Fig. 2. As seen in **Panel A**, morphine induced a decrease in pH from Pre values in rats that were injected with vehicle 30 min following administration of morphine (values are those 15 min after injection of vehicle, **M30:D15**) and again 60 min after administration of morphine (values are those 15 min after the second injection of vehicle, which occurred at time point 30 min, **M60:D45**). As also seen in **Panel A**, the first and second injections of D-CYSdime caused a pronounced recovery of pH. As seen in **Panel B**, the sustained increases in CO_2_ elicited by morphine in vehicle-treated rats were markedly reduced in rats that received the injections of D-CYSdime. As seen in **Panels C** and **D**, the sustained morphine-induced decreases in pO_2_ and SO_2_ that were observed in vehicle-injected rats were markedly reduced in rats that received D-CYSdime.

### 3.4. Reversal of the effects of morphine on A-a gradient by D-CYSee and D-CYSdime

The calculated A-a gradient values before and following administration of morphine (2 mg/kg/kg, IV) and following two injections of D-CYSee (100 μmol/kg, IV) or D-CYSdime (50 μmol/kg, IV) are shown in Fig. 3. As seen in **Panels A** and **B**, the administration of morphine elicited long-lasting elevations in A-a gradient in the separate groups of rats that received the two injections of vehicle (as measured 30 and 60 min after morphine administration) As also shown in these panels, the first and second injections of D-CYSee and D-CYSdime caused the recovery of A-a gradients toward pre-morphine values (injection 1) and to values somewhat equivalent to pre-morphine values (injection 2).

### 3.5. Summary of responses in ABG chemistry and A-a gradient

**Fig. 4.**
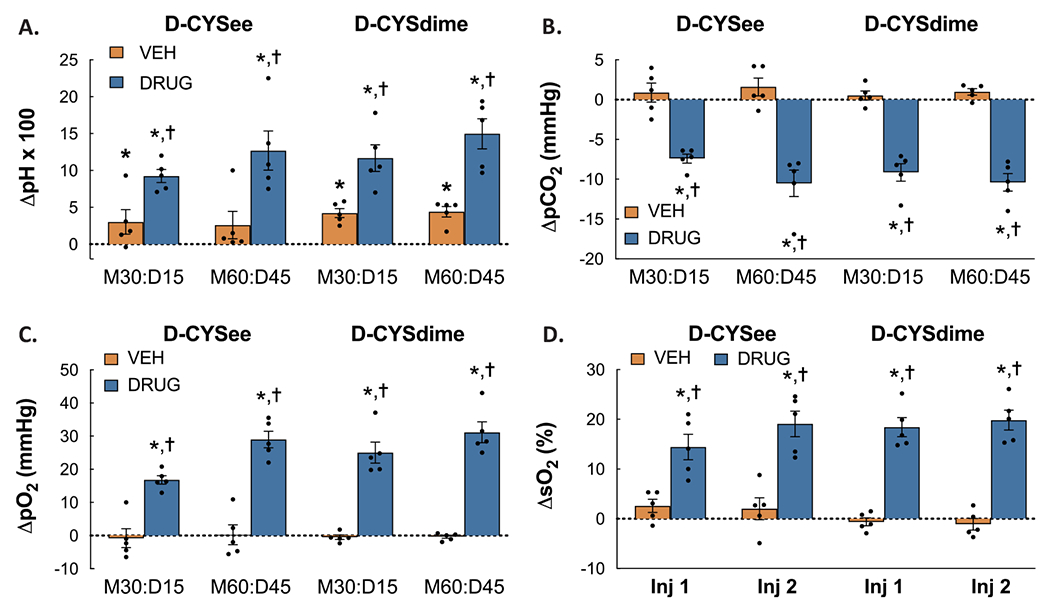
Arithmetic changes in pH (**Panel A**), pCO_2_ (**Panel B**), pO_2_ (**Panel C**) and sO_2_ (**Panel D**) from morphine values elicited by injections of vehicle, D-CYSee, or D-CYSdime. The data are presented as mean ± SEM. There were 5 rats in each group. * *P* < significant difference from Pre values. ^†^*P* < 0.05, D-CYSdime versus vehicle.

The arithmetic (delta) changes in pH, pCO_2_, pO_2_ and sO_2_ elicited by the two injections of D-CYSee or D-CYSdime in rats that were administered morphine are shown in Fig. 4. As is evident, both D-CYSee and D-CYSdime reversed the deleterious effects of morphine on pH, pCO_2_, pO_2_ and sO_2_ (**Panels A-D**, respectively). The arithmetic (delta) changes in A-a gradient produced by the injections of D-CYSee or D-CYSdime in the rats that were treated with morphine are summarized in **Panel C** of Fig. 3. Both D-CYSee and D-CYSdime profoundly reversed the deleterious effects of morphine on A-a gradient in these rats.

## 4. Discussion

This study demonstrates that D-CYSee and D-CYSdime produce a rapid and long-term reversal of the deleterious actions of morphine on ABG chemistry (i.e., reductions in pH, pO_2_ and sO_2_, and elevations in pCO_2_), and A-a gradient (i.e., opioid-induced responses indicative of ventilation-perfusion mismatch) in isoflurane-anesthetized rats that were not implanted with a tracheal tube to bypass the upper airway. First, these findings suggest that D-thiolesters improve ABG chemistry by enhancing minute ventilation and improving gas exchange in the lungs. This is consistent with our evidence that D-CYSee (unpublished findings) and D-CYSdime (Gaston et al., 2021) overcome the actions of morphine on breathing in conscious Sprague Dawley rats. Second, unlike L-CYSee (Mendoza et al., 2013), it appears that neither D-CYSee nor D-CYSdime compromise patency-diameter of the upper airway in morphine-treated rats. As such, the ability of thiolesters to promote an increase in airway resistance is dependent on stereospecific processes, whereas the ability to overcome the actions of morphine on ventilatory parameters, ABG chemistry and A-a gradient, is not. The administration of morphine elicits pronounced effects on ventilation in isoflurane-anesthetized humans (Drummond et al., 2013) and rats (Mendoza et al., 2013). The question as to whether the effects of morphine are exaggerated, unchanged, or inhibited under isoflurane anesthesia has not, to our knowledge, been ascertained. In our past studies and present data, we have found that the changes in ventilation and ABG chemistry elicited by a 2 mg/kg dose of morphine in isoflurane-anesthetized rats (Mendoza et al., 2013, present study) are equivalent to those elicited by a 10 mg/kg dose in unanesthetized rats (May et al., 2013a; b; Baby et al., 2018; Gaston et al., 2021). While detailed dose-response studies are needed, it appears that the ventilatory depressant effects of morphine may be augmented under isoflurane anesthesia.

### 4.1. Changes in ABG chemistry and A-a gradient elicited by morphine

In agreement with Mendoza et al. (2013), morphine elicited sustained reductions in pH, pO_2_ and sO_2_, and elevations in pCO_2_, in isoflurane-anesthetized rats. Changes which are expected consequences of morphine-induced depression of breathing (Trescot et al., 2008; Dahan et al., 2010). As expected (Mendoza et al., 2013), morphine elevated A-a gradient values, suggestive of unusually lower pO_2_ in lung blood than in alveoli (Torda, 1981; Story, 1996). A reduction in pO_2_, without alterations in A-a gradient results from hypoventilation. Because the reductions in pO_2_ produced by morphine were associated by elevations in A-a gradient, morphine probably produced a mismatch of ventilation-perfusion or shunting within the lungs. Morphine may have elevated pulmonary artery pressure by direct opioid receptor-mediated mechanisms, and/or exacerbated the vasoconstriction elicited by the decreases in ventilation caused by morphine. As such, the reduced flow of arterial blood to alveoli is probably a key process involved in the mechanism by which morphine diminishes arterial pO_2_ in rats anesthetized with isoflurane. This would agree with findings that opioids elevate resistance in the pulmonary circulation in animals (Schurig et al., 1978; Hakim et al., 1992).

### 4.2. Processes by which the disturbances in ABG chemistry and A-a gradient produced by morphine may be reversed by D-CYSee and D-CYSdime

In regards to potential mechanisms by which D-CYSee and D-CYSdime overcome the deleterious actions of morphine, it is unlikely that D-CYSee and D-CYSdime directly interact with morphine in vivo (e. g., chelate the opioid, thereby removing it from the circulation) because neither L-cysteine nor L-cystine directly interact with morphine in solution (Nagamatsu et al., 1982). In vitro studies found that L-cysteine, L-glutathione and L-dithiothreitol at a concentration of 1 mM failed to modulate opioid receptor binding, and a very high concentration of the thiol compounds (20 mM) causes a dramatic reduction in binding (Cox et al., 1980). Based on the presumption that the rats used in our study (approximately 0.3 kg in weight) have approximately 20–22 ml of blood in circulation (Ringler and Dabich, 1979), the first injections of D-CYSee (100 μmol/kg) and D-CYSdime would, at the moment of administration, produce blood levels of 1.5 mM of these thiolesters (i.e., each rat received about 30 μmol of D-CYSee producing concentrations of 30 μmol/20 ml = 1.5 mM) and 0.75 mM of these thiolesters, respectively. Assuming the unlikely scenario of zero degradation of D-CYSee or D-CYSdime or their distribution into tissues, injection 2 of these thiolesters would raise plasma concentrations to 3.0 mM and 1.5 mM, respectively. Taken together with the minimal effects of D-cysteine and D-cystine, it is not probable that the above doses of the thiolesters overcame the actions of morphine via direct effects on plasma membrane proteins, including opioid receptors (Laragione et al., 2006). In consideration of the known peripheral and central sites of activity of morphine (Christie et al., 2000; Boom et al., 2012; Baby et al., 2018; Gillis et al., 2020), it seems likely that D-CYSee acts in the carotid bodies and/or relevant brainstem sites, such as the pre-Bötzinger complex, to overcome the deleterious actions of morphine in these structures. The ability of D-CYSee and D-CYSdime to overcome the increases in A-a gradient produced by morphine raises the possibility that the thiolesters reduce the increases in pulmonary vascular resistance resulting from the direct effects of morphine and/or those resulting from morphine-induced hypoxemia (Mendoza et al., 2013).

Morphine causes the redox conditions of neuroblastoma x glioma hybrid cells to become more oxidative (Polanco et al., 2009), and diminishes the concentrations of reduced glutathione in cultured epithelial cells (Macchia et al., 1999). Accordingly, L-CYSee may overturn the effects of morphine by increasing the concentrations of L-cysteine (Butterworth et al., 1993; Hobbs et al., 1993; Deneke, 2000), L-glutathione (Kimura and Kimura, 2004; Kimura, 2010), and H_2_S (Kimura, 2010) inside of cells. It is known that L-cysteine (Deneke, 2000; Métayer et al., 2008; Winterbourn et al., 2008), L-glutathione (Hill and Bhatnagar, 2007), and H_2_S (Peng et al., 2010) have redox actions in cells, and that H_2_S stimulates breathing via effects within the carotid bodies (Peng et al., 2010). With respect to the processes by which D-CYSee and D-CYSdime overcome the deleterious actions of morphine, we have found that D-CYSee (unpublished data) and D-CYSdime (Gaston et al., 2021) overcome the ventilatory depressant actions of morphine in freely-moving rats, whereas neither D-CYSee (unpublished observations) nor D-CYSdime (Gaston et al., 2021) attenuate morphine-induced antinociception and sedation. As such, it is not likely that the beneficial actions of these thiolesters involves direct antagonism of opioid receptors. At present, we do not know if the actions of D-CYSee involve conversion to D-cystine diethyl ester (i.e., by formation of disulfide bonds) or that the actions of D-CYSdime involve reduction to two entities of D-cysteine methyl ester. Therefore, the question as to how the monosulfide and disulfide thiolesters affect redox status is unanswered. Nonetheless, the molecular mechanisms by which D-CYSee and D-CYSdime exert their effects may involve the production of H_2_S since in some tissues it is known that D-amino acid oxidase converts D-cysteine to 3-mercaptopyruvate that is converted to H_2_S by 3-mercaptopyruvate sulfurtransferase (Shibuya and Kimura, 2013; Shibuya et al., 2013). Whether these processes take place in structures relevant to how D-CYSee and D-CYSdime modulate the deleterious actions of morphine on ABG chemistry and A-a gradient is yet to be determined, although, the possibility that D-CYSee and D-CYSdime interact with plasma membrane ion-channels, receptors, enzymes, and intracellular signaling proteins should not be discounted.

### 4.3. Relevance to control of upper airway resistance

The difference between the ability of L- and D-CYSee to promote morphine-induced closure/collapse of the upper airway may lead to a deeper knowledge about how L- and D-cysteine, and their metabolic pathways, control upper airway structures, such as the larynx and vocal folds. Obvious mechanisms for L-CYSee could involve (1) increased intracellular levels of L-cysteine altering redox status, thus inducing a more reductive state of upper airway structures (Métayer et al., 2008; Winterbourn and Hampton, 2008), (2) enhanced formation of glutathione (Kimura and Kimura, 2004; Kimura, 2010), which modulates cell signaling by redox actions and by protein S-glutathiolation (Hill and Bhatnagar, 2007), (3) enhanced formation of H_2_S (Kimura, 2010), (4) increased bioavailability of L-cysteine and also L-glutathione, which promotes intracellular formation of S-nitroso-L-cysteine and S-nitroso-L-glutathione, respectively, which enhance the S-nitrosylation status and activities of numerous intracellular proteins (Gaston et al., 2006; Marozkina and Gaston, 2012, 2020), and (5) conversion of L-CYSee/L-cysteine to biologically-active sulfenic, sulfinic and sulfonic acids (Jacob et al., 1953–1956; Rahman et al., 2006; Gupta and Carroll, 2014). Obvious mechanisms for D-CYSee could involve (1) increased intracellular levels of D-cysteine altering redox status, therefore inducing a more reductive state of upper airway structures, (2) enhanced formation of H_2_S (Shibuya and Kimura, 2013; Shibuya et al., 2013), and (3) increased bioavailability of D-cysteine promoting intracellular formation of S-nitroso-D-cysteine, which may enhance S-nitrosylation status, and therefore the activities of intracellular proteins.

### 4.4. Summary

This study provides evidence that D-CYSee and D-CYSdime reverse the deleterious actions of morphine on ABG chemistry and A-a gradient in rats anesthetized with isoflurane, and unlike with L-CYSee (Mendoza et al., 2013), these positive effects are not negated by obvious collapse/closure of the upper airway. The negative actions of L-CYSee on the patency of the upper airway suggests the potential involvement of L-cysteine and downstream metabolites in physiological/pathophysiological control of the patency of the vocal cords and larynx. While L-CYSee could be administered to overcome the negative effects of opioids on breathing in intubated patients during or following surgery (Taylor et al., 2005), it would be clearly preferable to have a thiolester that overcomes the effects of opioids on ABG chemistry without causing upper airway obstruction. As such, the improved pharmacological profiles of D-CYSee and D-CYSdime, include the reversal of the deleterious actions of morphine on breathing, ABG chemistry and A-a gradient without causing an increase in upper airway resistance or interference with the pain-relieving actions of the opioid (Gaston et al., 2021). This study in isoflurane-anesthetized rats showing that D-CYSee and D-CYSdime reverse the deleterious effects of morphine on ABG chemistry is supported by our recent findings that (a) D-CYSdime reverses the detrimental effects of morphine on ABG chemistry and A-a gradient in unanesthetized rats (Gaston et al., 2021), (b) the related thiolester, D-cystine diethyl ester, reverses the deleterious effects of morphine on ventilation (Gaston et al., 2021), and (c) D-CYSme reverses the effects of morphine on ventilatory parameters and ABG chemistry in unanesthetized rats (Getsy et al., unpublished observations).

## Figures and Tables

**Table 1:** Numbers of rats and body weights on the study groups.

	Study A		Study B		Study C		
	vehicle	D-CYSee	vehicle	D-CYSdime	vehicle	D-cysteine	D-cystine
Number of rats	5	5	5	5	5	5	5
Body weight, grams	318 ± 2	317 ± 2	321 ± 2	323 ± 2	323 ± 3	319 ± 2 g	321 ± 2 g
Dose	^a^Saline	100 μmol/kg	^a^Saline	50 μmol/kg	^a^Saline	100 μmol/kg	50 μmol/kg

a The intravenous volume of saline (vehicle) administered to all rats was 100 μL/100 g body weight.

The data are presented as mean ± SEM. The were no differences in body weights between the groups in any study (P > 0.05 for all comparisons).

**Table 2:** Description of experimental protocols.

Time (min)	Post-thiol (min)	Procedure
−15		Pre-morphine arterial blood gas sample
0		Injection of morphine, 2 mg/kg, IV
15		Arterial blood gas sample
15	0	**Injection 1:-** Vehicle, 100 μL/kg, IV -CYSee, 100 μmol/kg, IV -CYSdime, 50 μmol/kg, IV -cysteine, 100 μmol/kg, IV or -cystine, 50 μmol/kg, IV
30	15	Arterial blood gas sample
45	30	**Injection 2** of vehicle or test thiols
60	45	Arterial blood gas sample

D-CYSee, D-cysteine ethyl ester; D-CYSdime, D-cystine dimethyl ester.

**Table 3:** Arterial blood-gas chemistry and Alveolar-arterial gradient in vehicle-treated rats.

		Sampling Periods			
Parameter	Treatment	Pre	Vehicle (pretreatment)	Dose 1 (vehicle)	Dose 2 (vehicle)
**pH**	Vehicle	7.443 ± 0.008	7.448 ± 0.011	7.442 ± 0.007	7.445 ± 0.010
**pCO_2_**	Vehicle	35.5 ± 0.3	35.6 ± 0.4	35.4 ± 0.4	35.3 ± 0.5
**pO_2_**	Vehicle	93.3 ± 0.9	93.6 ± 0.8	93.2 ± 0.7	93.1 ± 1.0
**sO_2_**	Vehicle	99.1 ± 0.6	99.2 ± 0.4	99.1 ± 0.5	99.0 ± 0.6
**A-a**	Vehicle	17.2 ± 0.8	17.0 ± 0.9	17.3 ± 0.7	17.5 ± 0.8

The data are presented as mean ± SEM. There were 5 rats in each group. The were no between time differences for any parameter (P > 0.05, for all comparisons).

**Table 4:** Arterial blood-gas chemistry and Alveolar-arterial gradients.

		Sampling Periods			
Parameter	Treatment	Pre	Morphine	Dose 1	Dose 2
**pH**	Vehicle	7.449 ± 0.005	7.326 ± 0.012 *	7.321 ± 0.015 *	7.324 ± 0.014 *
	D-cysteine	7.447 ± 0.006	7.327 ± 0.14 *	7.322 ± 0.008 *	7.327 ± 0.15 *
	D-cystine	7.446 ± 0.006	7.325 ± 0.011 *	7.328 ± 0.013 *	7.331 ± 0.013 *
**pCO_2_**	Vehicle	35.5 ± 0.2	45.7 ± 0.6 *	45.7 ± 0.4 *	45.8 ± 0.5 *
	D-cysteine	35.8 ± 0.2	46.3 ± 1.1 *	46.5 ± 0.8*	46.6 ± 0.6*
	D-cystine	36.0 ± 0.3	45.6 ± 1.0 *	45.4 ± 1.0*	42.1 ± 1.3 *,†
**pO_2_**	Vehicle	92.9 ± 0.8	69.7 ± 0.9 *	69.2 ± 0.7 *	70.7 ± 1.6 *
	D-cysteine	93.5 ± 0.8	68.9 ± 1.0 *	69.0 ± 1.1 *	69.6 ± 1.0 *
	D-cystine	92.7 ± 0.8	68.8 ± 2.7 *	69.2 ± 2.3 *	79.9 ± 2.1 *,†
**sO_2_**	Vehicle	100.2 ± 0.7	83.2 ± 0.6 *	83.1 ± 0.5 *	81.8 ± 1.1 *
	D-cysteine	100.5 ± 1.4	82.7 ± 0.7 *	81.3 ± 1.1 *	81.7 ± 0.6 *
	D-cystine	98.9 ± 0.5	81.4 ± 1.3 *	81.1 ± 1.2*	87.7 ± 0.4 *,†
**A-a**	Vehicle	17.4 ± 0.7	29.3 ± 0.5 *	29.7 ± 2 *	28.2 ± 1.9 *
	D-cysteine	16.4 ± 0.7	29.4 ± 2.0 *	29.1 ± 1.6*	28.3 ± 1.1 *
	D-cystine	17.0 ± 1.0	30.2 ± 2.2 *	30.1 ± 1.7*	23.0 ± 1.1 *,†

The data are presented as mean ± SEM. There were 5 rats in each group.

* P < significant effect of morphine.

† P < 0.05, Dose 2 drug versus vehicle.

## Data Availability

The sets of data that were produced by the experiments described in this manuscript will be freely available when requested of the author for correspondence.
